# Deciphering Fc-mediated Antiviral Antibody Functions in Animal Models

**DOI:** 10.3389/fimmu.2019.01602

**Published:** 2019-07-17

**Authors:** Alan L. Schmaljohn, Chiara Orlandi, George K. Lewis

**Affiliations:** ^1^Department of Microbiology and Immunology, University of Maryland School of Medicine, Baltimore, MD, United States; ^2^Division of Vaccine Research, Institute of Human Virology, University of Maryland School of Medicine, Baltimore, MD, United States

**Keywords:** virus, antibody, Fc, FcR, neutralization, ADCC, animal models

## Abstract

Longstanding discordances and enigmas persist as to the specificities and other properties of antibodies (Abs) most effective in preventing or limiting many viral infections in mammals; in turn, failure to decipher key complexities has added to headwinds for both Ab-based therapeutic approaches and rational vaccine design. More recently, experimental approaches have emerged—and continue to emerge—for discerning the functional role of Ab structure, especially the Fc portion of antibody, in combating viral infections *in vivo*. A wide range of *in vitro* measures of antibody activity, from neutralization to antibody-dependent cell mediated cytotoxicity (ADCC)—each of these terms representing only an operational notion defined by the particulars of a given assay—are poised for assignment of both relevance and reliability in forecasting outcomes of infection. Of the several emergent technical opportunities for clarity, attention here is drawn to three realms: the increasing array of known modifications that can be engineered into Abs to affect their *in vivo* activities; the improvement of murine models involving knockouts and knock-ins of host genes including Fc receptors; and the development of additional virological design tools to differentiate Abs that act primarily by inhibiting viral entry from antibodies that mainly target viral antigens (Ags) on cell surfaces. To illustrate some of the opportunities with either zoonotic (emerging, spillover) or ancient human-adapted viruses, we draw examples from a wide range of viruses that affect humans.

## Introduction

The network of interactions between virus and host is not only complicated, it represents a complex adaptive system of which Ab-mediated immunity is only one important part. Despite the bewildering complexity, some useful generalizations have emerged: *in vivo veritas*; or in a colloquial tautology used in reference to viral vaccines and therapies, *the only correlate of protection is protection*. Direct testing of effectiveness in human trials is the ideal benchmark for licensure of vaccines and therapies for human use; however, in cases for which human testing is unfeasible or unethical (1, 2), indirect rationales for product licensure must be established on the basis of animal models. That is, where human health is the predominant ultimate concern of research and development, and is the standard benchmark of its relevance, the most meaningful *in vitro* assays along with non-human models of disease are sometimes necessary surrogates for human efficacy trials. And while *in vitro* assays can be highly useful as possible statistical correlates of protection (3), they can also be poor reflections of complex realities: witness the abundant examples in which neutralization, binding titer, or hemagglutination-inhibition assays can be inadequate at best, misleading at worst (4–6). The fullness of what we wish to know about antiviral Abs is to be found in how Abs limit or sometimes exacerbate virus-precipitated disease in the body of an animal.

The scientific narrative on immunity to microbial pathogens has proceeded in waves, with peaks and troughs of emphasis on phagocytic cells, Abs, T cells, innate immunity, regulatory signaling, genomic analyses of immune repertoires, mechanisms of pathogen evasion of host responses, and so forth. Confounding the shifts in perceived importance of various aspects of immunity, there are differences in understanding of operational terms and their acronyms; a few of them used in this manuscript, and their intended meaning, are shown in Box 1. It is in this context, and with recognition that there already exist excellent recent reviews on discrete aspects of FcR-dependent antiviral immunity (8–16), that we aspire to offer a brief and possibly more holistic view of just one important aspect of virus-host interactions: the interactions between Abs, viral Ags, FcR, and FcR-bearing cells. We share in the anticipation and excitement of how emerging technologies may offer new experimental insights into complex processes that were previously suspected but unapproachable.

Box 1 **A brief guide to some fraught language.**

• **Neutralization:** (virus neutralization) An operational term typically referring to an observed Ab-dependent decrease in viral infectivity, gene product (antigen or tag), genome, spread, or other phenomenon in a particular *in vitro* assay.

• **ADCC:** Ab-dependent cell mediated cytotoxicity, a collective and operational (assay-defined) term rooted in many possible, varied, and nonredundant *in vitro* assays that measure *FeR-dependent activities facilitated by FeR-bearing cells* with readouts such as: target cell lysis; phagocytosis; trogocytosis; NK cell activation; granzyme release; or *ex vivo* FeR binding.

• **CMC:** Ab-dependent, complement-mediated cytotoxicity, typically referring to direct or indirect measurement of lysis of antigen-bearing cells in the presence of specific Ab along with heat-labile proteins known or presumed to execute the full complement cascade. Related assays but requiring addition of FeR-bearing cells include CDCC (complement-dependent cell-mediated cytotoxicity) and CDCP (complement-dependent cell-mediated phagocytosis)

• **Protection:** Here, this sometimes-ambiguous term refers to any of several favorable outcomes: (1) prevention of viral infection (“sterile” immunity); (2) post-infection control of viral load, with mitigation of acute disease (with or without viral clearance); or (3) in the case of latent or persistent infection, sustained remission of symptoms along with reduced viral load and diminished transmission.

                                                                                                                                                            -----

^*^A common language, with agreement upon the meaning of terms, is often missing from discussions of Ab-mediated immunity to viruses. A few important terms are shown here, as used in this manuscript. Background discussions of neutralization and ADCC have been provided previously (4, 5), *and CDCP elsewhere* (7).

## Overarching Questions

**Figure 1:**
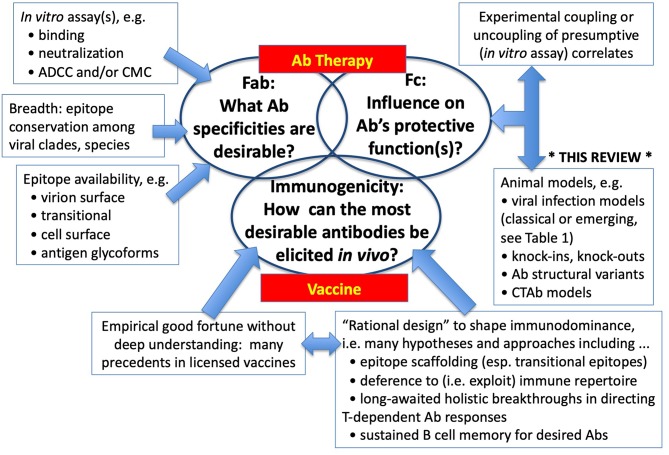
Vaccines and Therapies: Central Questions in Ab-mediated Resistance to Viral Infections. Where empiricism and conventional strategies have not led to effective Ab-based therapies or vaccines, investigators turn to deeper understanding of: (1) the paratope (epitope-binding moiety) on the Fab; (2) the biological function-amplifying structures of Ig molecules located mostly in Fc; and (3) the unsolved complexities of how to construct vaccine Ags and microenvironments (e.g., adjuvants, cytokines) that induce Abs mirroring those most desirable.

Some viruses yielded long ago to empirical approaches to vaccines and Ab therapies, and those who led such progress (e.g., Jenner, Pasteur, Theiler, Salk, Sabin, Hilleman) are due tremendous credit for their insights, inventiveness, boldness, and dogged determination. Many other viruses have not surrendered so easily to either serendipity or brilliance, and in the more advanced examples, promising vaccines or Ab therapies have not yet completed their costly and uncertain journeys to licensure. It is the intractable and previously orphaned problems at which research is now directed. Restricting attention here to Ab-mediated immunity to viral infections, three major and interrelated questions arise on the path to vaccines and therapies (Figure 1). What Ab specificities are responsible for protection and are most desirable for their breadth and safety? What other characteristics of Abs are important for protection, especially in the Fc part of the molecule? And when these answers are known, how might vaccine be configured to elicit the most desirable specificities and types of Abs? The latter questions of immunogenicity and immunodominance have proven problematic for the diverse human population, and rational shaping of immune responses (e.g., fine specificities, types, durability) remains perhaps the greatest challenge of immunology. Here, we focus on experimental approaches to the precursor questions of what *kinds* of Ab response are desirable, and more specifically how hypotheses drawn from provisional *in vitro* correlates of protection might withstand the test of *in vivo* veracity.

## The Virus-Ab Encounter

In previous reviews, we probed in some depth the matter of what *in vitro* virus neutralization is and is not, and how it does—and sometimes does not—align with an Ab's capacity to prevent or mitigate viral disease *in vivo* (4). We emphasized the redundancy of protective mechanisms typified by cell-targeting antibodies (CTAbs), i.e., those Abs (neutralizing or not) that mark virus-infected cells for interaction with various populations of Fc receptor (FcR)-bearing cells as well as complement (5). Many of the complexities previously noted, along with the kinds of protective functions that may or may not require FcR interactions, are summarized in Box 2). To simplify the narrative in this manuscript, the term “FcR” is used as shorthand for Fc gamma receptor (FcγR) unless otherwise specified. For those steeped in the large array of known FcR on immune effector cells as well as non-classical Fc-binding receptor homologs on a wider array of cells, we can only acknowledge the choice of brevity over an even greater narrative complexity, and point toward some of the many reviews available [e.g., reference (18). and citations in tables therein]. In like fashion, for the sake of brevity and “simplicity,” emphasis is on Fc's of IgG molecules despite the many important uncertainties about the antiviral, interfering, or synergizing roles of IgM, IgA, and even IgE in host immunity to viruses (19).

**Box 2:** Structure-function considerations in antiviral antibodies that may confer protection^*^.

**“Fc-independent” Ab-binding is sufficient. Fe serves only half-life and valency**	**“Fc-dependent” Ab-binding is necessary but insufficient. Fe is required for one or more functions**
• “**Neutralization**” i.e., Abs that act solely as antagonists of viral binding, entry, or launch as in many common assays • **Aggregation** of virions to functionally suppress numbers of infectious units. • **Antagonism of viral assembly**, e.g., competitive inhibitors of trimmer formations or required cleavages. • **Antagonism of viral release** processes, e.g., anti-neuraminidase • **Antagonism of viral “virulence factors**” that otherwise exacerbate disease, promote intercellular spread, or aid transmission	**Opsonization:** Abs that exploit FeR to redirect infectious virions to insusceptible cells, e.g., neutrophils. • **Cell-targeting antibodies (CTAb)** that require Fe receptor (FeR) interaction for manifestation of antiviral effect. - **ADCC** as measured (17) for example by: direct lysis of infected cells; trogocytosis (RFADCC); phagocytosis; granzyme release/signaling by NK - **Complement activation:** lytic cascade; other pleotropic effects of partial complement activation • many other potential interactions

* *See Box 1 for meaning of the term “Protection” in this manuscript. For discussions and additional references on these phenomena, refer to past reviews (4, 5)*.

**Figure 2:**
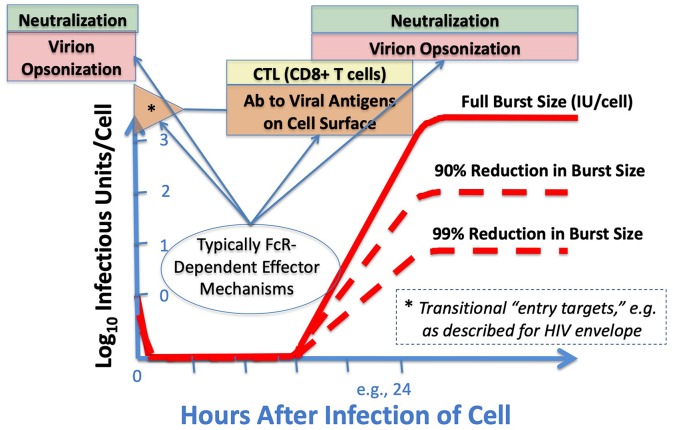
Adaptive immunity at the cellular level, and windows of opportunity. As virus enters cells (here at a multiplicity of infection around 1 infectious unit [IU] per cell, thus non-infectious particles at even higher ratios), disassembles, and then makes new proteins and genomes on the way to making more virions, the targets available to the immune system change. Conventionally, “neutralizing Abs” inactivate or sequester virus extracellularly, either before infection or as virus emerges. FcR-bearing cells can facilitate such extracellular clearance when the FcR-bearing cells are virus-resistant, and additional proteins—such as those of the complement cascade—can augment this opsonization. Before and during viral replication, either T cells (recognizing MHC-associated peptides) or cell-targeting Abs (CTAbs, recognizing emergent and pre-assembly proteins) can disrupt cell integrity and thereby diminish the viral yield per cell (burst size) by many-fold; the sparing of uninfected cells accrues exponentially. In some cases (well-described with HIV), the entering virions display new and early CTAb targets (*) as the viral spike rearranges coincident with receptor engagement. Emphasis in this manuscript is upon recent and emerging experimental tools to decipher the *in vivo* effects of Fc-FcR interactions that result in protection, including those involving Abs shown to score positively with *in vitro* neutralization assays, ADCC assays, or both.

To remind readers who are not steeped in virology, some fundamental features of virus-cell interactions in the context of adaptive immune responses are illustrated (Figure 2) to draw attention to the importance of viral replication cycle, the time-and-location distinctions between where Ab-dependent neutralization and Ab-dependent cell-targeting may occur in the cycle, and the rationales by which parts of the immune system mitigate rather than prevent infection. The special case of early, entry-associated targets for ADCC, best described with HIV, was covered in previous reviews (17, 20–23). Parenthetically, we affirm the complementary and interactive natures of Ab and T cell immunity, but that larger topic is not considered here. Moreover, Figure 2 depicts only the events at a single-cell level, and reserves for elsewhere a discussion of the larger complexities of localized and distant viral spread in the infected host, viral persistence, latency, and biological systems that drive the ultimate outcomes of host survival and viral spread to new hosts.

## Parallels With Tumor Models

In the realm of CTAb, expectations with antiviral Abs are at least partially informed by the literature on Ab-mediated suppression or elimination of tumor cell growth *in vivo*. In fact, the human data with Ab therapy designed to eliminate cells—or otherwise interact with cell surfaces to achieve a biological effect—are more advanced in oncology and autoimmunity than they are in virology, resulting in many more licensed Ab therapies for human use (12, 14, 24, 25). In the case of tumor cell elimination, functions such as ADCC, complement-mediated cytolysis (CMC), and monocyte/macrophage-mediated killing—all categories of activity that are well-dissected yet poorly understood—are appreciated as major immune mechanisms to be considered and manipulated. Routinely, developers seek to optimize Fc in order to maximize therapeutic effect and, when a proinflammatory response is undesirable, to minimize unhelpful inflammation in clinical trials. Similar clinical endeavors in human virology have been unfeasible or unethical. Nevertheless, where judged relevant, tumor models will be reflected in our considerations of how Abs act against virus-infected cells in Fc-dependent fashions.

## Some Past Challenges in Deciphering the Role of Fc in Animal Models

Overwhelmingly, immunological data in non-human species have come from mouse models, where inbred mouse strains and myriad research reagents allow complex cell and Ab transfers. In virology, however, mice tend to be wholly or partially resistant to infection and disease caused by human pathogens of greatest interest; a compromise is sometimes found by serial passage of virus in mice to achieve some semblance of human disease and protection. For many years, even when there was a palatable model of viral disease in mice, and early data suggested an important role for Fc in Ab-mediated resistance to certain of those viruses (4), data were generally unconvincing in assigning clear relevance to Fc because of technical limits, to wit: the necessary panels of virus-specific monoclonal antibodies (MAbs) having identical paratopes (Ag combining sites) but different Fc moieties were then unachievable. Consequently, a preponderance of evidence that murine MAbs of IgG2a subtype were generally most protective (especially for CTAbs) were less than definitive; Fab and F(ab)_2_ fragments of Abs were almost exclusively non-protective (or poorly so) but were not directly comparable to intact Ab because the fragments (neutralizing *in vitro* or not) had short half-lives *in vivo*; attempts to deplete FcR-bearing cells *in vivo* were confounded by the overall toxicity and secondary effects of such depletions, so that truly appropriate controls were lacking; complement (C') depletion of mice typically left the protective capacity of whole Abs intact, but redundant mechanisms (ADCC as well as CMC) and incomplete C' depletion remained possibilities. Moreover, when cross-species transfers of Abs were made (e.g., human Abs into mice or non-human primates [NHP], mouse Abs into guinea pigs or NHP), positive protective results (e.g. Ab-mediated protection) were useful but negative results were fraught [not only do anti-Abs arise in a few days to eliminate xeno-Abs, but also the Fc-FcR interactions across species are problematic at best (26)].

## Advances in Virology and Viral Pathogenesis – Beyond Passage-Adapted Viruses to Engineered or Chimeric Challenge Viruses, or Virus-Susceptible “Humanized” Mice

In search of an animal model for viral disease, classical adaptation of virus by serial passage sometimes fails repeatedly, and the accumulating evidence on the nature of species barriers for any given virus may sometimes suggest that adaptation through mutation and selective pressure is highly improbable. At best, and through the lens of product licensure, Golding writes, “The establishment of animal models predictive of vaccine effectiveness in humans has been fraught with difficulties with low success rate to date.”(1) Today, however, there are many ways to: (1) refashion genes of a human virus to become more likely to cause infection and disease in non-human species; (2) refashion genes of unrelated viral pathogens (e.g., ordinarily restricted to mouse or NHP) to express and incorporate presumptive “protective Ags” of human pathogens, in order to test mechanisms of immunity targeted against those antigens; (3) render a non-human species (especially mice) more human-like in susceptibility through engraftment of human cells, or through specific gene knock-ins (e.g., of human receptors for virus) and knock-outs (e.g., of host-range resistance factors such as interferon). A few examples and references are given in Table 1.

**Table 1:** Classical and emerging approaches for deciphering Fc-mediated antiviral Ab functions in animal models.

**General experimental approach, tool**	**Selected (Representative) examples**
Where virus cannot cause meaningful infection or disease in a known animal model, *make a new virus*	simian/human HIV (SHIV) (27, 28); reconstructed (and controversial) influenza viruses (29, 30); surrogate live virus (e.g., VSV, vaccinia) expressing Ag from virus-of-interest
Where virus cannot cause meaningful infection or disease in a known animal model, *make a new animal*	Transduced (31) or transgenic (32) mice expressing MERS-CoV receptor; transgenic mice susceptible to hepatitis C (33, 34); multigenetic variants (35)
Identify and exploit naturally uncoupled (i.e., either/or) targets for anti-virion vs. anti-cell Abs	Alphaviruses, poxviruses, flaviviruses [reviewed in (4)]
For a given viral epitope of interest, construct *panel of MAbs having same paratope, different Fc isotypes*.	melanoma cells in mice (36); non-IgG isotypes underexplored; most viral systems reported to date lacked matching paratopes (4, 5, 37, 38)
For a given viral MAb of interest, construct *Ab variants ablated or augmented in FcR binding, complement activation, or other activity*.	tumor immunology (39, 40); influenza virus (6, 8); West Nile virus (38);
Limit examination to CTAbs by expressing one or more viral Ags on cells, then measuring *immune clearance of viral Ag-expressing cells*	HIV expressing influenza HA (41, 42)
Determine whether engineered (knockout) *mice lacking one or more FcR still retain protection by Ab*	West Nile virus (38), influenza (6), HIV (41)
Examine Ab-mediated *protection in mice lacking murine FcR but expressing one or more (knock-in) human FcR*	Mice so designed (26)
Examine Ab-mediated protection in (knockout) *mice lacking discrete FcR-bearing cell lineages*	Presaged in mice newly designed with knock-in human FcR to examine tumor immunity (26)

## CTAbs and the Utility of Cells that Express Viral Ags

As noted previously (5), the role of Fc-dependent Ab activities on viral clearance *in vivo* has too often been subordinated to an unfruitful “either-or” argument about relative importance of “neutralizing” vs. “non-neutralizing” Abs, when in fact many (but not all) neutralizing monoclonal MAbs (nMAbs) are also potent cell-targeting MAbs (CT-MAbs) see Boxes 1, 2. To separate the effects of conventional nAbs from Abs that also (or exclusively) exert protective effects on viral Ag-expressing cells, the literature on tumor immunology provides useful guides in work that is already well-described and continuously evolving. For example, it is well-established that: (a) MAbs against tumor antigens (expressed on cell surfaces) can direct the elimination of antigen-expressing cells by mechanisms that require appropriate interactions between Fc and FcR (12, 36, 43); (b) anti-tumor activities (both *in vitro* and *in vivo*) can be either improved or diminished by making changes in the Fc portions of MAbs, including glycosylation (39, 44, 45); (c) complement as well as FcR can have a role in cell elimination, and experimental tools to untangle the two are improving (7, 15, 46–49); and (d) MAb interactions with inhibitory FcR can be important determinants of outcome, undermining protective effects and promoting Ag internalization (36, 50–53).

**Figure 3:**
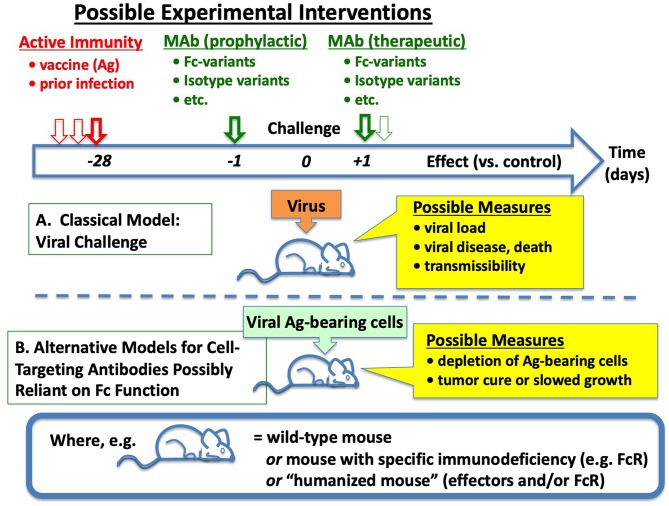
Simplified Overview of Emerging Approaches for Discernment of Roles of Fc in Ab-mediated Immunity to Viruses. **(A)** In classical models, experimental animals (often mice) are challenged on day 0 with infectious virus, preceded (or sometimes followed) by intervention with active or passive immunization. **(B)** To disambiguate the collection of phenomena that only affect virions and their spread, and examine more closely the requirements for effective Fc-mediated effects against viral Ag-expressing cells, one can challenge mice instead with cells engineered to express viral Ags, somewhat mimicking prior models of tumor immunotherapy. See text and references for many of the potential complexities and opportunities in each step, including modified Abs, and highly modified mice.

Obviously enough, many of the same experiments could be replicated—and rational improvements in antiviral Ab efficacies (and vaccines) possibly suggested—by testing the capacities of different Abs (in animal models with different FcR) to eliminate cells constitutively expressing viral membrane Ags of interest, as illustrated in Figure 3B. Indeed, such work has already begun (41, 42).

In addition to deeper understandings and new directions, practical rewards could arise from the establishment of model systems designed to find mechanistic correlates between *in vitro* assays and *in vivo* elimination of viral Ag-expressing cells: genuinely predictive models could facilitate bridging studies from animal models to human efficacy, otherwise a barrier for licensure of vaccines that require invocation of the “Animal Rule” (1).

## Cumulative, Synergistic, and Antagonistic Variables in Discernment

**Figure 4:**
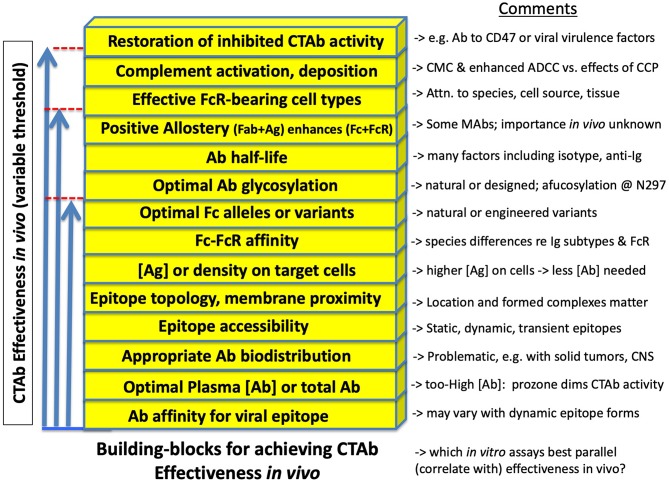
Concepts represented as building blocks for effectiveness of cell-targeting Abs in immunity to viral infections. See text for details. Many factors—each with its own quantitative or qualitative dimension—contribute additively or synergistically to reaching a threshold of antiviral activity that meets a standard of protective efficacy (as defined in Box 1). The threshold is not a constant, but varies by individual, host species, virus biotype, dose and route of infection, etc. In short, the figure is intended to illustrate that Fc-mediated aspects of Ab-mediated immunity are interrelated parts of a complex adaptive system that arose during co-evolution of host and virus. To adequately exploit these concepts with vaccines or therapeutic Abs requires either empirical good fortune (the key to almost all licensed vaccines) or understandings that allow rational improvements in design. In the search for statistical or mechanistic correlates of protection, assays that combine several of these factors may be favored.

As illustrated in Figure 3, animal models may be employed to examine effectiveness of Ab given at various times before (prophylactically) or after (therapeutically) the cognate Ag, in this case viral antigen either on virions or cells. Interpretations of outcomes are in some respects straightforward. However, behind the superficial simplicity of Abs including CTAb that may evoke Fc-dependent antiviral effector function, there exists a complex array of binding and signaling events, each with its own quantitative and qualitative dimensions. To simplify, these are illustrated in Figure 4 as building blocks required to reach a threshold of activity that is itself variable. Some of these are more widely familiar than others, as described next.

Proceeding from the bottommost variable illustrated in Figure 4, the importance of *Ab affinity for its cognate viral epitope* is well-known and foundational in immunology. As a general rule, higher affinity Abs are more active in most *in vitro assays*, and in protection. However, affinity is less an intrinsic property of Ab-epitope interaction than a measurement that can depend (over several orders of magnitude) upon epitope framework, conformation, epitope masking, and dynamic changes (rearrangements) that can occur for example when viral spikes encounter receptors, proteases, or pH changes (4, 5, 54).

Ab *concentration* is a familiar variable insofar as a concentration threshold is typically observed, below which a given Ab (monoclonal or polyclonal) is apparently ineffectual. Related, however, is the role of *Ab biodistribution* (its concentration at the necessary site of activity) which may undermine Ab effectiveness by providing insufficient concentration in critical sites including solid tumors, brain, retina, intestine, lung, and testes (55). Experimentally, as in animal models described here, there is a particular hazard at the opposite end of Ab concentration, i.e., the impulse to empirically screen for Ab effectiveness using high-dose treatments is contraindicated (as a singular approach) by the phenomenon of high-dose *prozone*, which presumably results from a diminution of the formation of complexes necessary for robust Fc-FcR interactions (56), akin to high-dose prozone in classical immunoprecipitation reactions. Another explanation offered [outside the binding-valency model (56)] is that the encounter between high Ab dose and high Ag burden results in an exhaustion of complement components (especially C2 and C4) with the consequence of diminished overall antibody effectiveness *in vivo* (15). In experimental animal models, a range of Ab doses is preferred.

*Epitope accessibility* refers to the longstanding observation, recapitulated in different ways with many viral Ags (4, 5, 54), that some viral epitopes are available to Ab (and thus to Fc-dependent immune effector mechanisms) only at selected times (or transiently) during the viral entry and growth cycle. We used the word “cryptic” in 1983 to describe epitopes that appear to be inaccessible on intact virions yet available on virus-infected cells; but “availability” may in other instances be an inadequate oversimplification of the dynamic nature of viral spike proteins.

*Epitope topology and membrane proximity* can determine spatial relationships and steric hindrances that favor one type of Fc-mediated effector function over another, illustrated for example in the development of therapeutic anti-CD20 Abs (40, 57) and strongly implied with a panel of influenza-specific MAbs (6). Some current data suggest that: “… complement-dependent cytotoxicity and Ab-dependent cellular cytotoxicity favored a membrane-proximal epitope, whereas Ab dependent cellular phagocytosis favored an epitope positioned further away.” (57) More broadly, the favored (optimal) configuration for a given effector function may vary with different Ag-Ab pairs, exemplified by counter-examples in which HIV-specific MAbs against the “membrane proximal region” of envelope may have negligible effector function (22). Generalizations are difficult, as they are confounded by mechanistic differences between *in vitro* assays (17); *in vivo* efficacy will be decisive.

*Ag concentration (or density) on target cells* is a factor in shifting the apparent effectiveness of Ab, i.e., there is an inverse relationship between Ag expression on target cells and the amount of Ab required to meet a threshold of Fc-dependent activity. Part of anti-CD20 therapeutic efficacy is attributed to high expression of CD20 on tumor cells (40), and it was recently shown that increased expression of target Ag on cells is one way to improve Fc-mediated cell clearance in mice otherwise compromised by persistent viral infection (58).

*Fc-FcR affinity*. We begin with a quote in a recent paper from the Ravetch group, leaders in the field of Fc-FcR interactions, and murine models with which to explore biological significance: “An antibody**'**s Fc domain**'**s relative affinity for the activating and inhibitory Fc**γ**R, called the A/I ratio, can determine its functional output, and is directly correlated to therapeutic efficacy *in vivo*. This has spawned recent efforts to engineer Abs with enhanced activating Fc**γ**R affinity.” (26) Embedded in much of their work is the directly observed or implied importance not only of isotype (36) but also species matches in establishing Fc-FcR affinity: human, mouse, and non-human primate (NHP) FcR are non-equivalent in binding to any given Ab (typically, human IgG1 is the chosen type), and nomenclature of FcR in the various species is a poor guide to Ab affinity and function.

The current scientific literature is rich with the recognition of naturally occurring *Fc and FcR alleles or variants* (19, 59), followed by structural redesigns of Ab molecules to optimize *Ab glycosylation* (8, 12, 39) or *Ab half-life* (60). The details are outside the scope of this manuscript, but as with other variables cited above, we call attention to the caution with which the Fc-dependent possibilities or limitations of a single MAb may be viewed during the course of experiments.

*Allosteric change in Fc* is a phenomenon that remains incompletely resolved in terms of biological significance. However, improved tools in structural analysis restore the possibility that allostery is among the factors that may contribute additively or synergistically to Ab function. Thus, in a subset of MAbs and presumably in a subset of natural polyclonal Abs, the binding of Fab to its cognate epitope results in *allosteric change in Fc*, which in turn promotes higher affinity between Fc and FcR than is observed in the absence of Ag (61, 62). The implication is that, depending upon the assay, *in vitro* results may be an inadequate predictor of Fc-mediated functional activity of Ab. A different kind of allostery is seen when two MAbs synergize on the basis of how Fab binding to one part of an Ag molecule promotes binding of a second MAb recognizing a different epitope on the same Ag (63). Newer animal models may add clarity to the functional importance of such allosteric interactions in Ab-mediated protection against viruses.

The importance of *effective FcR-bearing cell types* should not be underestimated, especially in cross-species transfers, such as human Ig transferred into NHP or mice. The complexities are several, due not only to interspecies differences in FcR affinity for any given MAb, but interspecies differences in the cell types on which various FcR are found (26).

In virological circles, *complement activation and complement deposition* have received diminishing attention, presumably because ablation of complement (in murine models) tends to leave intact the protective antiviral capacity of an Ab (4, 5). However, the dismissal of complement is likely imprudent, as antibody therapies against human tumors show important additive and augmenting effects mediated by complement (39, 48, 49, 64). As signaled in Figure 4, attention must be paid to the additive and synergistic Fc-mediated effects of Abs, as these cumulatively determine whether a threshold is reached in which an Ab is effective *in vivo*.

The potential for *restoration of inhibited CTAb activity* is an emerging opportunity for understanding and improving the protective capacities of CTAb against viruses. Once again, tumor immunology has led the way, with anti-CD47 Abs already in clinical trials, and showing clinical promise by way of dampening a “don't eat me” signal that otherwise spares tumor cells from destruction by monocytes and macrophages (65, 66). Many poxviruses explicitly express CD47 homologs, and other complex viruses such as herpesviruses express homologs of immunomodulatory proteins (67). The potential for anti-CD47 to shift the threshold for antiviral attention has not escaped notice (58), but this may be only the first of many opportunities to counteract known (67) and perhaps unknown viral proteins that undermine host immune responses including Fc-dependent activities. To venture a testable hypothesis, this could be part of the mechanism by which a herpesvirus subunit (HZ/su), consisting of one of the family of herpesvirus proteins (gE) that binds Fc (68), serves as an effective vaccine (68): by evoking Abs that restore otherwise-inhibited and FcR-dependent CTAb activity.

## Concluding Remarks

While some elements of Ab-mediated antiviral immunity appear to be largely or completely independent of Fc function, others are highly reliant on Fc in order to exert biological effects that register as “protective” activity (Boxes 1, 2). Such Abs (CTAbs) share with a number of therapeutic anti-cancer Abs the aim of arresting or destroying cells recognized by such Abs, and mechanistic relevance can be gleaned from the extensive research and clinical trials with anti-tumor CTAbs. However, the efficacies of antiviral CTAbs are differently complex due to both the typical incapacities to obtain human protection data and the longstanding problems inherent with classical animal models of viral disease. More recently, newer approaches (Table 1) have allowed increasing compatibility between a given virus (or its cell-expressed antigen), a susceptible animal model, Fc-FcR interactions, populations of FcR-bearing effector cells, etc. This brief review is intended to highlight and cite some of the recent literature that first points toward an almost bewildering complexity in the factors that intrude upon the subset of protective antiviral mechanisms that are Fc-dependent, and then to some feasible approaches to achieve clarity. Most likely, for a given virus with its unique structure and biology, and its unique pathogenetic profile in a chosen animal model, some Fc-dependent Ab-directed mechanisms will prove more reliably important than others. Moreover, each of the concepts illustrated in Figure 4 is meant here to be understood as scalable in its importance, but rarely if ever sufficient by itself to achieve a threshold that achieves enough efficacy to adequately suppress viral replication and prevent viral disease. It is most encouraging that experimental tools, including those shown in Table 1, are emerging to affirm or refute the truth and biological importance of concepts previously mired in the complexities. To an increasing degree, rational selection and design of optimally effective MAbs (and the means to elicit them with vaccines) will inform antiviral research. Similarly, refinement and selection will continue to improve for *in vitro* assays that are not only statistical correlates but mechanistic correlates of protection. And still, the final proofs will continue to be an empirical matter of finding what is both safe and effective.

## Author Contributions

AS, CO, and GL shared and discussed in lab meetings over a period of many months in the development of perspectives, emphases, and cited references for this manuscript. AS crafted the primary draft, which was improved, and approved by CO and GL.

### Conflict of Interest Statement

The authors declare that the research was conducted in the absence of any commercial or financial relationships that could be construed as a potential conflict of interest.
